# Driving force of value reversal in Chinese overleveraged firms: The mechanism and path of private placement

**DOI:** 10.1371/journal.pone.0303544

**Published:** 2024-05-13

**Authors:** Xin Song, Xiaodi Liu, Huiyu Chen

**Affiliations:** University of Shanghai for Science and Technology, Shanghai, China; University of Almeria: Universidad de Almeria, SPAIN

## Abstract

To stimulate economic growth, China has launched multiple economic stimulus plans in recent years, intensifying corporate debt financing and subsequently elevating the leverage levels. Addressing and effectively reducing the leverage levels of our country’s enterprises has emerged as a pressing issue in the trajectory of our economic development. This paper primarily investigates the drivers, pathways, and mechanisms for reversing the over-leveraged values of enterprises. Key findings include: (1) Excessive indebtedness exerts a negative impact on corporate value, with the suppressing effect intensifying as the degree of over-leverage increases; (2) Over-leveraged enterprises can effectively decrease their debt levels and enhance their value through private placement. Further research suggests that this mechanism operates by amplifying the operational leverage of over-leveraged enterprises post private placement and alleviating financing constraints, thereby elevating corporate value. (3) Compared to non-state-owned enterprises, state-owned enterprises exhibit higher levels of indebtedness. Among over-leveraged firms, enhancements in corporate governance and increased investment efficiency can positively transform corporate value. This study offers valuable insights for the ongoing supply-side structural reforms and governance guidance from the regulatory bodies.

## 1. Introduction

High leverage rates stand as one of the paramount issues that economic development must confront. In pursuit of sustained and healthy economic progression, transitioning effectively from rapid to high-quality growth, China has rolled out a series of developmental economic policies. The Central Economic Work Conference in December 2015 clearly put forward that the core task of supply-side structural reform is " address overcapacity, reduce inventory, deleverage, lower costs, and bolster areas of weakness ", which is the first time that China has proposed deleveraging. In 2016, The Central Economic Work Conference emphasized that reducing the leverage ratio of enterprises was the top priority. In July 2017, the National Financial Work Conference further clarified the focus on strengthening the deleveraging of state-owned enterprises and standardizing the government’s borrowing behavior. In 2018, when The People’s Bank of China issued the RRR reduction policy, it clearly pointed out that the targeted RRR reduction funds did not support the projects of "fake equity and real debt " and "zombie companies ", the funds from the RRR reduction should be used for loans to small and micro enterprises, further highlighting the policy objectives of physical and structural deleveraging. Statistics from CSMAR indicate that excessively leveraged enterprises constitute 46.3% of the total enterprises. While indebtedness can bridge corporate capital shortfalls, alleviate financial strains, and introduce leveraging effects, excessive indebtedness augments the financial risks, increases financing constraints, jeopardizes continuous healthy corporate progression, and might even, in extreme cases, trigger capital chain disruptions leading to corporate bankruptcy and restructuring [1]. Before 2018, corporate deleveraging predominantly employed a "blanket" approach. While this method considerably reduced corporate debt levels, but the strict deleveraging policy may make it difficult for enterprises to obtain financing, which limits the investment and expansion of enterprises and affects the economic growth rate. At the same time, the over tightening deleveraging policy may lead to the instability of the financial market, and even systemic financial risks, causing serious impact on the entire financial system [2]. Therefore, overly strict " one-size-fits-all approach " deleveraging may have serious negative effects on the economy and society, which requires policymakers to maintain prudence and balance in the implementation of deleveraging policies to avoid unnecessary economic and social losses. To address the deleveraging issue more scientifically, the State Council, the National Financial Work Conference, the Central Committee for Financial and Economic Affairs have successively put forward a series of policy objectives for structural deleveraging. "Structural" refers to adopting different and targeted methods for financial risks in different fields and markets in China, giving priority to problems that threaten macroeconomic stability or easily lead to systemic risks, this policy is in line with the current situation of China’s industrial adjustment, transformation and upgrading [3]. Contrasted against the traditional "blanket" method, "structural deleveraging" is more rational, advocating for targeted debt financing reduction where enterprises, contingent upon their industry and individual circumstances, adopt varying strategies [4]. In June 2018, the People’s Bank of China announced a targeted reduction of 0.5% in the reserve requirement ratio, intended to make the " debt-for-equity swap " market-oriented and legal, fully highlighting the policy direction of structural deleveraging.

At present, the research on deleveraging in China is mainly divided into macro-economic level and micro enterprise level. From the macro level, China’s corporate debt continued to rise in the past few years, especially the huge debt scale of state-owned enterprises and local government financing platforms [5]. Dou Wei and others believe that China’s shadow banking system is large in scale, including informal financing methods such as trust, financial management and private equity funds. These shadow banking institutions provide a lot of implicit financing, which increases the opacity and risk of the financial system [6]. Today, China’s deleveraging has experienced from policy guidance to strengthening supervision, and then to steadily promote structural deleveraging. As a result, China’s deleveraging policy has achieved remarkable results. With the strengthening of financial supervision and the control of financial risks, the stability of China’s financial system has been improved, and some financial institutions and projects with serious risks have been disposed of, which has strengthened the stability and anti-risk ability of the financial system. From the micro level, some scholars believe that deleveraging of over indebted enterprises can effectively enhance the value of enterprises and improve the utilization rate of funds [7]. In reality, private placement is an important way to balance the capital structure of enterprises and reduce the debt level [8]. When the leverage ratio of an enterprise is excessively high, its risks and burdens will increase accordingly. In this context, these enterprises may seek private placement as a strategy to adjust their capital structure and reduce financial pressure. However, can this strategy really help enterprises reverse the shortage of funds and operating difficulties? Which channels have promoted this transformation? This paper makes a systematic exploration and research around these problems. Through empirical research, it is found that excessive debt has a significant negative effect on enterprise value. The higher the debt level, the stronger the inhibition effect on enterprise value. In order to improve the capital structure, over indebted enterprises tend to reduce the debt level by private placement. After the private placement, the enterprise value can be effectively improved by increasing the operating leverage and reducing the financing constraints. In addition, state-owned enterprises are more indebted than non-state-owned enterprises. In over indebted enterprises, improving corporate governance and investment efficiency is an effective way to improve enterprise value.

The primary contributions of this paper are as follows: (1) It enriches the literature on corporate value. While existing studies on factors influencing corporate value primarily center around internal innovation, investment efficiency, and operational status, there’s a notable scarcity of research approaching the relationship between corporate over-leverage and value from the perspective of private placement. (2) The results of this study augment the research framework on the value of balanced capital structure behaviors and offer novel insights and avenues for further exploring how private placement impacts corporate value. (3) By examining how companies utilize private placement to enhance their existing capital structures and its subsequent influence on corporate value, this paper provides pragmatic guidance for the ongoing supply-side structural reforms.

## 2. Theoretical analysis and research hypotheses

In modern corporate enterprises, the separation of ownership and management rights is very common. According to the principal-agent theory, in order to maximize their own interests and speculative psychology, managers will use more internal information they have to blindly expand the scale of the enterprise and increase their control power [9]. To swiftly achieve these objectives, enterprises gather substantial funds in the short term. The loose credit environment will also reduce the financing cost of enterprises and promote enterprises to expand their own scale and investment. Moderate debt can realize leverage effect, so as to improve the profitability and value of enterprises and reduce the cost of capital [10]. Since judicious and appropriate leveraging can yield the leverage effect and reduce the capital cost of the enterprise, and given that national tax regulations permit pre-tax deductions for debt, this "tax shield" functionality of debt offers tax benefits to enterprises. As an effective way for enterprises to expand rapidly and solve the shortage of funds in the short term, borrowing can promote rapid economic growth. However, when enterprises use debt financing to expand investment and production, the leverage effect will drive the performance growth of enterprises when the income is higher than the debt cost, but when the actual investment income is lower than the financing cost, the rise of leverage will hinder the development of enterprises and reduce the value of enterprises [11].

In reality, the increase of capital demand of enterprises usually leads to the increase of debt level. According to trade-off theory, when the level of debt continuously escalates and exceeds the target level, the tax benefits conferred by the debt are insufficient to offset the associated losses. This leads to a situation where marginal costs exceed marginal benefits, resulting in a reduction in enterprise value. Liu and Yang selected a sample of 9,860 Chinese A-share listed companies from 2001 to 2010 to investigate the influence of debt structure on company growth under a debt system threshold. They discovered that borrowing negatively affects company value when the leverage ratio is below 20% or exceeds 70% [12]. Consequently, effectively reducing the leverage level can enhance enterprise value and improve the investment efficiency of enterprises [13]. Ma and Zhu (2020) also analyzed large-sample data from Chinese manufacturing enterprises from 1998 to 2013, examining the impacts of deleveraging and optimal capital structure on the productivity of real enterprises [14]. They argue that deleveraging in over-indebted enterprises is conducive to improving enterprise productivity.

On the other hand, when businesses opt for short-term debt financing, they are obligated to repay the loans within a shorter timeframe, necessitating greater liquidity requirements. This, to some extent, can hinder the growth of the enterprise [15]. In comparison to short-term debt, long-term debt generally involves larger amounts and extended repayment periods. And the financing costs associated with long-term debt are higher, exerting greater repayment pressures on the businesses. When an enterprise has too much debt, its financial risk increases. This means that enterprises need to pay more interest and bear higher debt repayment pressure. If an enterprise fails to repay its debts on time, it may face the risk of default, which will damage the reputation of the enterprise, lead to the rise of financing costs, and further aggravate the financial pressure [16]. In order to repay high debts and meet the requirements of bank loans, enterprises may increase the investment in mortgaged fixed assets and reduce the investment in innovation activities such as R&D, thus limiting the innovation ability of enterprises [17]. When the debt maturity of an enterprise approaches, the enterprise will face greater repayment pressure. In extreme cases, this repayment pressure may lead to the rupture of the capital chain, and ultimately lead to the bankruptcy and reorganization of the enterprise [18].

As China’s economy transitions from rapid expansion to high-quality development, the overall economic size grows, and the general economic trend enters a phase of moderate-to-low growth. Excessive leveraging can result in a marginal EBIT (Earnings Before Interest and Taxes) rate that’s lower than the interest rate on the debt, thereby exerting a negative influence on corporate value [19]. When enterprises accumulate excessive debt, even mild external shocks may lead to tightening of debt constraints and bring difficulties to enterprises’ refinancing in the capital market. Even if they manage to raise funds, creditors often embed various protective and restrictive clauses within the loan agreements. As the financial resources of enterprises are constrained, enterprises may have to give up some good investment opportunities. In the face of high debt ratio, enterprises will even sell assets at low prices to repay debts, which is likely to cause a negative impact on the asset value in the enterprise’s balance sheet and reduce the value of the enterprise’s net assets, which will lead to investors’ loss of confidence and further reduce the enterprise’s value [20]. Based on this, we propose Hypothesis 1 and Hypothesis 2.

**H1:** Excessive debt decreases firm value.
**H2:** The greater the extent of a firm’s excessive debt, the stronger its suppressive effect on firm value.

Private placement is one of the significant methods for equity refinancing of enterprises. According to statistics from Wind Data, ever since the China Securities Regulatory Commission enacted the "Measures for the Management of Securities Issuance by Listed Companies" in 2006, which introduced the concept of private placement financing, private placement has consistently constituted over 90% of the refinancing market. Owing to its numerous advantages such as low financing costs, straightforward application and implementation procedures, and ease of operation, private placement has gradually become the preferred method for equity refinancing for corporations. Companies engage in private placement for various reasons, including project financing, capital operations, and supplementing working capital.

For companies with excessive debt, reducing debt is a common strategy to reduce financial leverage, but the implementation of this strategy usually requires enterprises to have sufficient cash flow to meet possible capital needs [21]. Over indebted enterprises often need a lot of funds to repay their debts or raise funds to reduce their debts through private placement [22]. However, excessive financial leverage makes it difficult for enterprises to have enough funds to repay debts. It is almost impossible to achieve the purpose of deleveraging through debt repayment. Even if they can repay debts, it will also have a serious negative impact on the production and operation of enterprises in the future. Therefore, enterprises with excessive debt are more likely to reduce the asset liability ratio through private placement. Enterprises raise cash through private placement, which can be used to repay debts or other investments [23]. This strategy will help reduce the debt level of the enterprise, reduce the financial pressure, and will not reduce the disposable cash flow of the enterprise. Based on the above, the following Hypothesis 3 is proposed.

**H3:** Over-leveraged firms, aiming to optimize their capital structure, tend to favor "private placement" as a strategy to reduce debt levels.

## 3. Data and research design

### 3.1 Sample selection and data source

This study selects A-share listed companies from 2010 to 2020 as the research sample and performs the following treatments on the initial sample: (1) Excluding the number of companies in the financial sector. (2) Excluding the number of ST and *ST listed companies. (3) Excluding the number of delisted companies. (4) Excluding the number of companies with missing data. After these exclusions, we obtained 11,096 company-year observations. Because there may be a two-way causal relationship between independent variables and dependent variables, leading to the inaccuracy of model estimation, this paper treats the independent variables and control variables in the model with a lag period, and moves the independent variables and control variables backward by one time unit, so as to make the direction of causal relationship more clear, better capture the influence of independent variables on dependent variables, and reduce the confusion of causal relationship. Additionally, to mitigate the impact of outliers on regression results, we perform a Winsorize tail-trimming on all continuous variables at the 1% and 99% levels. The internal control index data used in this study is sourced from the DIB (DiBo) database, while all other data come from the CSMAR and WIND databases.

### 3.2 Model construction and variable definitions

#### 3.2.1 Measuring over-leverage

There are three commonly used methods to measure over-leverage: (1) Subtracting the target leverage ratio from the actual book leverage ratio of the company. (2) Subtracting the median or mean industry leverage ratio of that year from the actual book leverage ratio of the company. (3) The interest expense when a company’s leverage achieves the maximum tax advantage divided by the actual interest expense [24]. Given that the target leverage ratio is determined by a comprehensive set of factors, this study adopts the first method to measure over-leverage. Specifically, drawing on the approach of Denis and McKeon (2021), we use the following model to predict a firm’s target leverage ratio.


$${Levb}_{i,t}=\alpha_{0}{+\alpha }_{1}{Soe}_{i,t-1}+\alpha_{2}{Roa}_{i,t-1}+\alpha_{3}{Ind\_levb}_{i,t-1}+\alpha_{4}{Growth}_{i,t-1}+\alpha_{5}{Fata}_{i,t-1}+\alpha_{6}{Size}_{i,t-1}+\alpha_{7}{First}_{i,t-1}$$

The dependent variable *Levb* represents the target leverage ratio. The independent variables capture factors affecting leverage ratios for firms in China: *Soe* represents the ownership structure, *Roa* refers to profitability, measured by the ratio of net income to year-end total assets. *Ind_levb* represents the median industry leverage ratio. *Growth* represents the total asset growth rate. *Fata* is the proportion of fixed assets. *Size* represents the firm size, measured using the natural logarithm of assets. *First* is the shareholding ratio of the largest shareholder.

#### 3.2.2 Model design

To test Hypothesis 1 and Hypothesis 2, which examine the impact of corporate over-leverage on firm value, we construct regression Model (1).

$${Evar}_{i,t}=a_{0}{+a}_{1}{Exlevb}_{i,t-1}+\sum a_{i}{Control}_{i,t-1}+\sum Ind+\sum Year+\varepsilon_{i,t}$$ (1)

Where *i* denotes the firm and *t* denotes the year. The dependent variable *Evar* represents the firm value, measured by the Economic Value Added (EVA) rate. In Model (1), The independent variable *Exlevb* represents over-leverage, comprises two components: *Exlevb_dum* and *Exlevb_ratio*. *Exlevb_dum* is a dummy variable, using "0" or "1" to indicate the presence or absence of over-leverage in the firm. *Exlevb_ratio* is the difference between the actual book leverage ratio (*Lev*) and the target leverage ratio (*Levb*). A larger value of *Exlevb_ratio* indicates a higher degree of over-leverage in the firm. *Control* denotes control variables: *Cash* represents the ratio of monetary capital, *Size* is firm size, *Soe* represents the ownership structure, and *First* is he shareholding ratio of the largest shareholder.

To test Hypothesis 3, which investigates if over-leveraged firms aim to improve their capital structure by private placement, we develop regression Model (2).


$${Treat}_{i,t}=a_{0}{+a}_{1}{Exlevb}_{i,t-1}+\sum a_{i}{Control}_{i,t-1}+\sum Ind+\sum Year+\varepsilon_{i,t}$$ (2)

*Treat* is a dummy variable that denotes whether a firm has conducted a private placement. *Exlevb* and *Control* is defined as above. Additionally, we include year and industry fixed effects. Detailed definitions and descriptions of the above variables are provided in Table 1.

**Table 1:** Variable names and definitions.

Symbol	Variable Name	Variable Definition
Evar	Firm Value	Evar = Economic added value / Total investment
Treat	Private Placement	Dummy variable, 1 for private placement, 0 otherwise
Exlevb_dum	Firm is Over-leveraged	Dummy variable, 1 if Lev > Levb, 0 otherwise
Exlevb_ratio	Extent of Over-leverage	Exlevb_ratio = Lev—Levb
Lev	Book Leverage Ratio	Lev = Year-end total liabilities / Total assets
Levb	Target Leverage Ratio	Based on the regression results from Denis and McKeon (2012)
Cash	Monetary Fund Ratio	Cash = Monetary funds / Total assets
Size	Firm Size	Size = Natural logarithm of assets
First	Largest Shareholder Share	Share percentage of the largest shareholder
Soe	Ownership Type	Dummy variable, 1 for state-owned firms, 0 for private firms
Year	Year Fixed Effects	Controls for yearly factors
Ind	Industry Fixed Effects	Controls for industry factors

Since there may be a two-way causal relationship between the independent variables and dependent variables of the Models (1) and (2), in order to reduce the impact of this two-way causal relationship, we treat the independent variables and control variables in the model with a lag period. Lag processing is a common research method, which has been recognized by most scholars. The basis for adopting this research method comes from the causal inference principle and time series analysis method. Its purpose is to solve the confusion of causal relationship when there is a two-way causal relationship, and improve the accuracy and interpretability of the model. Furthermore, we conducted a variance inflation factor (VIF) test. The mean VIF results were consistently below 10 (see Table 2), indicating the absence of multicollinearity issues among the model’s variables.

**Table 2:** Variance inflation factor (VIF) test.

	Model (1)		Model (2)	
Variable	VIF	1/VIF	VIF	1/VIF
Exlevb_dum	1.09	0.913	2.08	0.481
Cash	1.13	0.884	3.06	0.327
Size	1.25	0.80	10.58	0.095
First	1.09	0.919	6.90	0.145
Soe	1.18	0.851	1.93	0.517
Mean VIF	1.15		4.91	

## 4. Empirical test results

### 4.1 Descriptive statistics of variables

Table 3 presents the descriptive statistics of the main variables used in this study. Based on the table data, the average leverage ratio for Chinese companies stands at 44%, with a minimum value of 4.95% and a maximum of 92%. The average target leverage ratio is 44.9%, ranging from 25% to 75.8%. Among all companies, 46.3% are over-leveraged, suggesting that over-leverage remains a prevalent issue in China. This observation aligns with the nation’s recent "deleveraging" policy initiatives. In Table 3, the Economic Value Added Rate (*EVAR*), which measures firm value, has a mean value of 0.164%, a minimum of -33.5%, and a maximum of 23.5%. This indicates significant variability in the Economic Value Added across different companies, setting the stage for subsequent analyses examining how over-leveraged companies balance their capital structure through equity enhancement and its impact on firm value.

**Table 3:** Descriptive statistics.

Variables	N	mean	sd	min	max
Evar	11,096	0.002	0.08	-0.335	0.235
Treat	11,096	0.138	0.345	0	1
Exlevb_dum	11,096	0.463	0.499	0	1
Exlevb_ratio	11,096	-0.01	0.174	-0.375	0.492
Lev	11,096	0.44	0.213	0.049	0.92
Levb	11,096	0.449	0.107	0.25	0.758
Cash	11,096	0.177	0.125	0.0132	0.65
Size	11,096	22.12	1.245	19.63	25.88
First	11,096	0.349	0.151	0.089	0.75
Soe	11,096	0.428	0.495	0	1

### 4.2 Main regression analysis

Table 4 presents the regression results of Model (1), illustrating the impact of over-leverage on firm value. The results in columns (1) and (2) reveal that there is a significant negative correlation at the 1% level between firms experiencing over-leverage and their value. Specifically, over-leverage tends to decrease firm value. The correlation coefficient stands at -0.019, indicating that for every 1 percentage point increase in the likelihood of a firm being over-leveraged, its value will decline by 0.019 percentage points. Columns (3) and (4) examine the effect of the degree of over-leverage on firm value. The regression outcomes indicate that the degree of over-leverage remains significantly negatively correlated with firm value at the 1% level, suggesting that the greater the extent of over-leverage, the stronger its suppressive effect on firm value. Excessive leverage hinders value creation for firms, sends signals of poor company management, and makes financing in the market more challenging. So which mechanisms can companies break this vicious cycle, improve their capital structure, and enhance their value?

**Table 4:** The impact of over-leverage on firm value.

Variable	(1)	(2)	(3)	(4)
	Evar	Evar	Evar	Evar
Exlevb_dum	-0.025***	-0.019***		
	(-14.27)	(-10.47)		
Exlevb_ratio			-0.087***	-0.062***
			(-17.43)	(-12.10)
Cash		0.112***		0.105***
		-16.02		-14.78
Size		0.011***		0.010***
		-14.27		-13.37
First		0.042***		0.044***
		-7.27		-7.51
Soe		-0.018***		-0.017***
		(-9.80)		(-9.16)
_cons	0.011***	-0.260***	-0.001	-0.253***
	-9.19	(-15.62)	(-1.37)	(-15.21)
Ind	control	control	control	control
Year	control	control	control	control
N	8475	8475	8475	8475

Note: ***, **, and * denote significance at the 1%, 5%, and 10% levels, respectively. Numbers in parentheses are t-values.

Table 5 displays the regression results of Model (2), examining whether over-leveraged firms engage in private placement to alter their existing capital structure. Columns (1) and (2) show the results of the Logit regression. Without accounting for other control variables, there is a significant positive correlation at the 1% level between over-leverage and private placement, with a correlation coefficient of 0.616. This implies that over-leveraged firms tend to raise funds through private placement to reduce their debt-to-asset ratio. Even after accounting for other control variables, over-leverage and private placement maintain a significant positive correlation at the 1% level. Furthermore, to account for the potential impact of certain time-invariant variables on the study results, we conduct a Hausman test to choose between fixed effects and random effects for Model (2). The p-value for the Hausman test is 0.00, leading us to reject the null hypothesis and adopt a fixed effects regression model. Columns (3) and (4) in Table 5 present the regression results of the fixed effects model, both without and with the consideration of control variables. After accounting for the control variables, there remains a significant positive correlation at the 1% level between over-leverage and firms conducting non-public stock offerings. This conclusion aligns with the Logit regression results, suggesting the research findings are fairly robust. Overall, the regression analyses from Tables 4 and 5 support Hypotheses 1 to 3.

**Table 5:** Private placement of over-leveraged firms.

Variable	(1)	(2)	(3)	(4)
	Treat	Treat	Treat	Treat
Exlevb_dum	0.616***	0.638***	1.017***	0.932***
	(10.08)	(9.90)	(8.50)	(7.45)
Cash		-1.166***		-4.827***
		(-4.26)		(-8.80)
Size		-0.112***		-0.739***
		(-3.99)		(-7.57)
First		-0.310		1.352
		(-1.46)		(1.88)
Soe		-0.422***		-0.932*
		(-6.29)		(-2.25)
_cons	-2.015***	0.923		
	(-43.37)	(1.51)		
Ind	control	control	control	control
Year	control	control	control	control
*N*	8475	8475	4188	4188

Note: ***, **, and * denote significance at the 1%, 5%, and 10% levels, respectively. Numbers in parentheses are t-values.

### 4.3 Robustness tests

To alleviate potential endogeneity issues in the model, this study has taken a lag of variables in the aforementioned tests. To ensure the empirical results’ reliability, we conducted the following series of robustness checks.

#### 4.3.1 Propensity score matching (PSM)

Common PSM techniques include nearest neighbor matching, radius matching, kernel matching, and Mahalanobis metric matching. This study employs the nearest neighbor 1-to-1 matching for its robustness test. Firstly, samples with a value of 1 for *Exlevb_dum* are defined as the "treatment group," while those with a value of 0 for *Exlevb_dum* are the "control group". Next, we select variables that might influence a firm’s excessive debt level as covariates. Covariates chosen in this study include the cash-to-assets ratio (*Cash*), firm size (*Size*), the shareholding ratio of the largest shareholder (*First*), and the nature of property rights (*Soe*). Finally, we run a Logit regression. The overall average treatment effects from Tables 6 and 7 indicate significant regression results after matching, consistent with the conclusions presented earlier in the text.

**Table 6:** Overall average treatment effect for Model (1).

Variable Sample	Treated	Controls	Difference	S.E.	T-stat
Evar Unmatched	-0.014	0.011	-0.025	0.002	-14.27***
ATT	-0.014	0.006	-0.02	0.002	-8.15***
ATU	0.011	-0.003	-0.014	——	——
ATE			-0.017	——	——

**Table 7:** Overall average treatment effect for Model (2).

Variable Sample	Treated	Controls	Difference	S.E.	T-stat
Treat Unmatched	0.198	0.118	0.08	0.008	10.24***
ATT	0.198	0.119	0.079	0.011	7.42 ***
ATU	0.118	0.203	0.085	——	——
ATE			0.082	——	——

**Fig 1:**
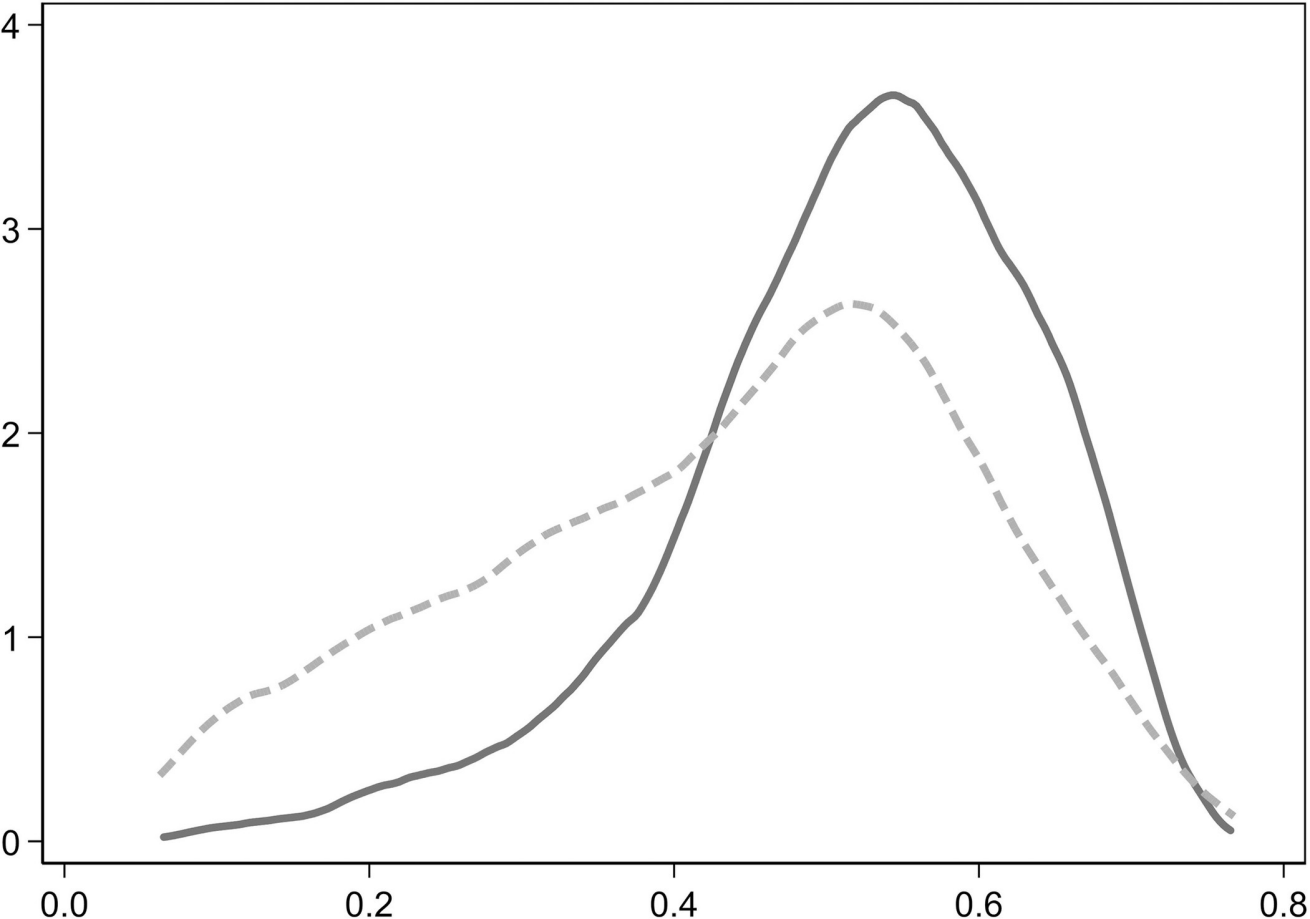
Density function graph before matching.

**Fig 2:**
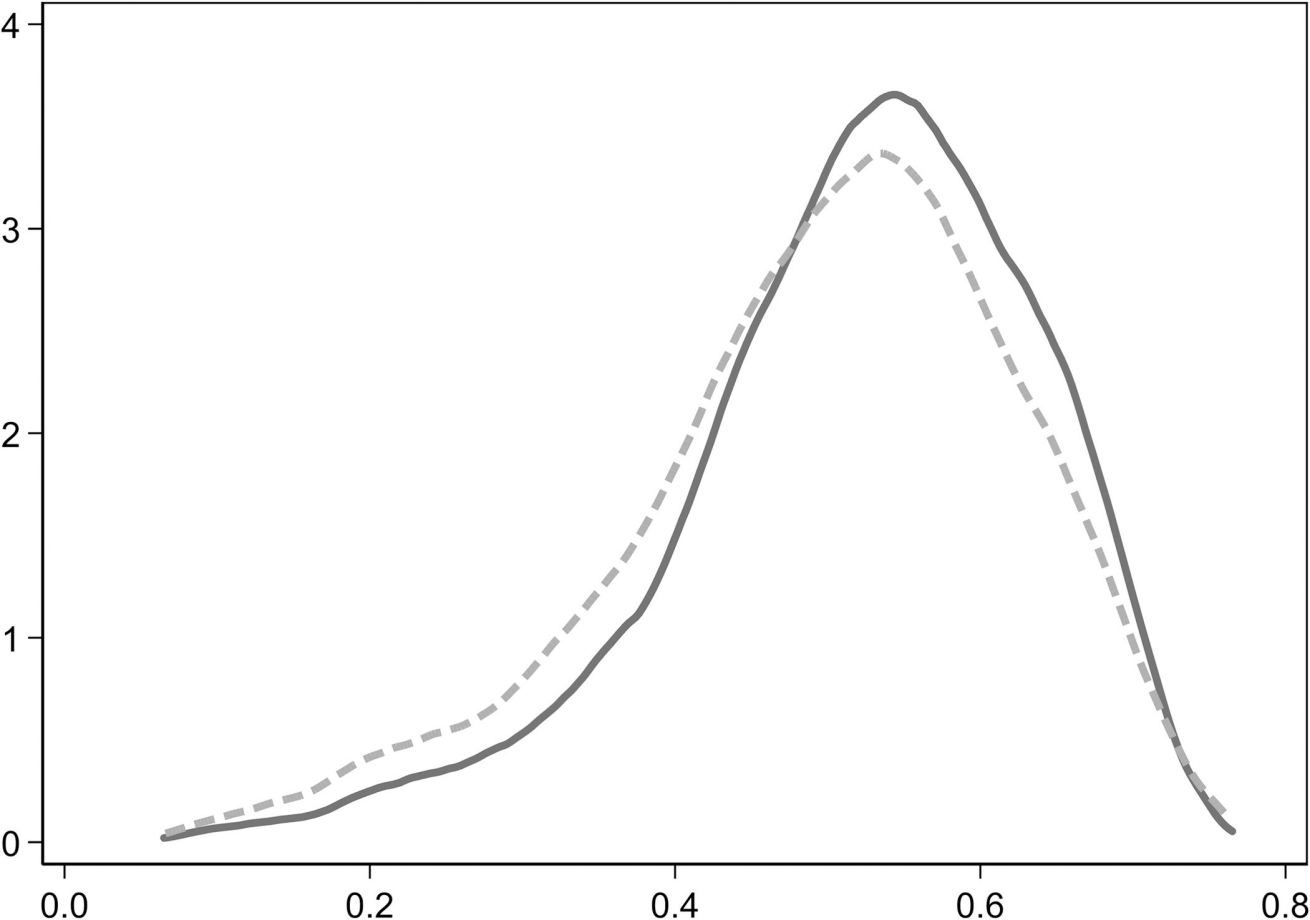
Density function graph after matching.

To assess whether the post-matching data is balanced and to ensure the quality of the match, thus making the matching results externally reliable, a balance test was performed based on the preliminary results. The test results show a reduction in the bias of the monetary fund ratio by 97.1%, a reduction in the bias of firm size by 80.7%, an increase in the bias of the first major shareholder’s shareholding ratio by 138.4%, and a reduction in the property rights bias by 91.0%. Before matching, there was a significant difference between the treatment group and the control group. After matching, while some differences in covariates remain between the two groups, the overall matching effect is satisfactory. Additionally, the matching results were visually presented. Figs 1 and 2 respectively depict the density function graphs of each variable before and after matching. The matching test results indicate that the differences between the treatment group and the control group have been substantially reduced after matching.

#### 4.3.2 Replace dependent variable

To enhance the credibility of the empirical findings, this study conducts a robustness test by altering the metrics used to measure the dependent variable. Replace the metric for enterprise value with the Tobin Q, calculated as follows: Tobin Q = Market value/Total assets. If the Tobin Q value is greater than 1, it indicates that the market value of the company exceeds its asset value; On the contrary, if the Tobin Q value is less than 1, it means that the market value of the company is lower than its asset value. Thus, examining Tobin Q allows for an indirect assessment of company value. And because Tobin Q value is determined by the market and is generally considered to be independent of the internal decision-making and financial status of the enterprise, it can be used as an exogenous variable to eliminate the endogeneity between asset liability ratio and enterprise value. Based on this, the model was regressed according to the previous steps, and the analysis results were found to be consistent.

#### 4.3.3 Switching control variables and controlling for chairperson characteristics

In Models (1) and (2), the firm size is represented by the natural logarithm of assets. In the robustness checks, this is replaced with the natural logarithm of the main business revenue to represent firm size. When reintroduced to the regression, the results remain consistent with previous findings. Additionally, the robustness checks control for individual characteristics of the company’s chairperson, including: education (*P_education*), gender (*P_gender*), and age (*P_age*). Keeping the other control variables constant, Models (1) and (2) are re-regressed, and the results remain consistent with previous findings.

#### 4.3.4 Ensuring companies have three years of listing experience

To avoid the potential influence of sporadic events on the regression conclusions and to further validate the robustness of the empirical results, data processing is carried out. Annual sample observations from the years 2018–2020 are removed to ensure that companies have at least three years of listing experience. Based on this, the models are re-regressed following the previous analysis steps, and the results remain consistent with prior findings.

## 5. Further analysis

### 5.1 The pathway through which private placement impact firm value

Based on the conclusions from our previous research, excessive debt can significantly negatively affect firm value. To mitigate the impact of excessive debt on firm value, firms tend to reduce debt levels by "equity infusion", thereby improving their capital structure and enhancing firm value. So, when excessively indebted firms undertake private placement, how do private placement enhance the value of over-indebted firms? What is the mechanism? The following will delve deeper into this question by constructing models and analyzing data.

There are two scenarios for a firm’s private placement. First is issuing shares at a discount to existing shareholders. After the issuance, these newly issued shares have a lock-up period of 36 months, during which they cannot be transferred. To ensure a rise in stock price during this lock-up period, shareholders will more actively supervise company operators, preventing moral hazard and adverse selection, thus promoting an increase in firm value [25]. From an operational perspective, an increase in corporate funds might lead the company to engage in short-term, high-return projects, thereby increasing operational leverage and boosting profitability, ultimately promoting firm value. However, some researchers have found that issuing directed shares to original shareholders can create a tunneling effect, reducing firm value [26]. The second scenario involves issuing shares to specific external investment institutions, which can introduce external investors and dilute the control of the original shareholders. Institutional investors play a crucial role in external corporate governance in the capital market. Compared to regular investors, they have stronger motivation and capability to oversee management [27]. After participating in private placement, strategic investment institutions tend to actively engage in corporate governance, supervising management, improving investment efficiency, and reducing operational costs, leading to value addition [28]. In contrast, financial investment institutions, driven by short-term profit-seeking motives, might not effectively supervise top executives, and might even prefer high-risk, high-return operations, thus increasing the operational leverage. Additionally, after conducting private placement, the financial status of over-indebted firms changes, producing a positive effect on firm value. Based on the aforementioned analysis, we further propose Hypothesis 4.

**H4:** Private placement enhance firm value by increasing operational leverage and altering the firm’s financial condition, producing a positive effect.

To test whether private placement have a positive impact on the value of over-indebted firms, we construct Model (3) for empirical analysis.


$${Evar}_{i,t}=a_{0}{+a}_{1}{Treat}_{i,t-1}+\sum a_{i}{Control}_{i,t-1}+\sum Ind+\sum Year+\varepsilon_{i,t}$$ (3)

Next, we employ a stepwise regression approach to examine the mediating effects.

#### 5.1.1 Testing the mediating effect of operational leverage

To examine the influence of changes in operational leverage on the firm value post private placement, this study employs the Altman Z-score as a measure for operational leverage. The calculation formula for *Z* is as follows.


$$Z=1.2X_{1}+1.4X_{2}+3.3X_{3}+0.6X_{4}+0.99X_{5}$$

$$X_{1}=\frac{net\;working\;capital}{total\;assets}=\frac{current\;assets-current\;liabilities}{total\;assets}$$

X_1_ reflects a company’s short-term debt repayment capacity. Generally, the higher this ratio, the stronger the company’s ability to meet its short-term liabilities.


$$X_{2}=\frac{ratained\;earnings}{total\;assets}=\frac{capital\;surplus+undistributed\;profits}{total\;assets}$$

X_2_ measures a company’s retained earnings. The larger the value, the greater the proportion of profits retained within the company.


$$X_{3}=\frac{earnings\;before\;interest\;and\;taxes}{total\;assets}=\frac{total\;proits+financial\;expenses}{total\;assets}$$

X_3_ measures the profitability of a company. The higher the value, the stronger the company’s ability to generate profits.


$$X_{4}=\frac{market\;value\;of\;shareholders\prime \;equity}{total\;liabilities}$$

X_4_ reflects the relative relationship between a company’s shareholder capital and creditor capital, showcasing the company’s capital structure. A higher value of this ratio indicates a lower risk of bankruptcy for the business.


$$X_{5}=\frac{sales\;revenue}{total\;assets}=\frac{operating\;revenue+other\;operating\;revenue}{total\;assets}$$

X_5_ reflects the company’s ability to generate sales. The higher the value, the more efficient the company’s asset utilization.

According to Altman’s research, when the Z-score is greater than or equal to 3, the company is unlikely to go bankrupt; if the Z-score is less than or equal to 1.8, the company is likely to go bankrupt. If the Z-score is between 1.8 and 3.0, it indicates that the company is in a gray zone, within which there is a 95% chance of bankruptcy within a year and a 70% chance within two years. Clearly, the smaller the Z-score, the more likely the company is to go bankrupt. Conversely, the larger the Z-score, the lesser the risk of bankruptcy for the company.

To verify the impact of operational leverage changes on the value of firms that private placement, Models (4) and (5) are established.


$$Z_{i,t-1}=b_{0}+b_{1}{Treat}_{i,t-1}+\sum b_{i}{Control}_{i,t-1}+\sum Ind+\sum Year+\varepsilon_{i,t}$$ (4)

$${Evar}_{i,t}=c_{0}+c_{1}{Treat}_{i,t-1}+c_{2}Z_{i,t-1}+\sum c_{i}{Control}_{i,t-1}+\sum Ind+\sum Year+\varepsilon_{i,t}$$ (5)

In Model (4), operational leverage (*Z*) is the dependent variable, and private placement (*Treat*) are the independent variable. In Model (5), firm value (*Evar*) is the dependent variable, operational leverage (*Z*) is the mediator variable, and private placement (*Treat*) are the independent variable. *Control* denotes control variables, including the ratio of monetary capital (*Cash*), firm size (*Size*), ownership structure (*Soe*), and the shareholding ratio of the largest shareholder (*First*). Over-leveraged companies, through private placement, aim to balance their capital structure by reducing financial leverage on one hand, and on the other hand, by attracting investments, they ensure that they have sufficient capital for their operations. The research investigates whether over-leveraged companies, after raising funds through private placement, increase their operational leverage. According to the high-risk, high-reward principle, companies with higher operational leverage tend to achieve higher value.

#### 5.1.2 Mediation analysis of financing constraints

In this study, we follow the approach of Hadlock and Pierce and employ the Sa index to gauge the financing constraints faced by businesses. The measurement method is outlined as follows.


$$Sa=0.043\times {Size}^{2}-0.737\times Size-0.04\times Age$$

*Age* represents the number of years a company has been listed. *Sa* is a reverse indicator, meaning that the greater the absolute value of the *Sa* index (*Sa_c = | Sa |*), the lower the level of financing constraints faced by the company.

To examine the impact of financing constraints on the value of overleveraged firms, we establish Models (6) and (7).


$${Sa\_c}_{i,t-1}=b_{0}+b_{1}{Treat}_{i,t-1}+\sum b_{i}{Control}_{i,t-1}+\sum Ind+\sum Year+\varepsilon_{i,t}$$ (6)

$${Evar}_{i,t}=c_{0}+c_{1}{Treat}_{i,t-1}+c_{2}{Sa\_c}_{i,t-1}+\sum c_{i}{Control}_{i,t-1}+\sum Ind+\sum Year+\varepsilon_{i,t}$$ (7)

Table 8 presents the empirical test results for the mediation effects of operating leverage and financing constraints. In column (1), we have the regression results for private placement and firm value, which show a significant positive correlation at the 5% significance level. This indicates that private placement has a positive impact on firm value. The correlation coefficient between the two is 0.008, meaning that a 1-percentage-point increase in private placement leads to a 0.008-percentage-point increase in firm value. Columns (2) and (3) present the mediation effects of operating leverage. In column (2), the results show that private placement is significantly positively correlated with operating leverage at the 5% significance level. In column (3), when both *Treat* and *Z* are added to the model, the correlation between private placement (*Treat*) and firm value (*Evar*) becomes insignificant, indicating a decrease in significance. Operating leverage remains significantly positively correlated with firm value at the 1% significance level. Therefore, in overleveraged firms, operating leverage plays a certain mediating role in the impact of private placement on firm value. Columns (4) and (5) present the empirical regression results for the mediation effects of financing constraints. In column (4), the regression results show that private placement in overleveraged firms is positively correlated with financing constraints. Since *Sa* is a reverse indicator, private placement reduces financing constraints. In column (5), private placement, financing constraints, and firm value are significantly positively correlated, indicating that financing constraints partially mediate the relationship, allowing firms to improve their firm value by reducing financing constraints.

**Table 8:** Mediation effects testing for operating leverage and financing constraints.

Variable	(1)	(2)	(3)	(4)	(5)
	Evar	Z	Evar	Sa_c	Evar
Treat	0.008**	0.559**	0.005	0.048**	0.009**
	(2.00)	(2.00)	(1.23)	(2.12)	(2.09)
Z			0.001***		
			(4.97)		
Sa_c					0.018***
					(3.20)
Cash	0.165***	4.675***	0.163***	0.428***	0.160***
	(12.69)	(5.45)	(12.16)	(6.09)	(12.16)
Size	0.008***	-0.203***	0.009***	1.128***	-0.013*
	(7.22)	(-2.71)	(7.72)	(184.15)	(-1.89)
First	0.055***	4.505***	0.043***	0.381***	0.049***
	(6.06)	(7.51)	(4.56)	(7.76)	(5.33)
Soe	-0.020***	-0.104	-0.021***	-0.259***	-0.016***
	(-7.30)	(-0.58)	(-7.55)	(-17.44)	(-5.28)
_cons	-0.232***	4.425***	-0.246***	-20.502***	0.148
	(-9.41)	(2.71)	(-9.75)	(-154.13)	(1.21)
Ind	control	control	control	control	control
Year	control	control	control	control	control
*N*	4294	4055	4054	4292	4259

#### 5.1.3 Other impact mechanisms

*(1) Corporate governance and firm value*. The empirical results presented above support the notion that over-leveraged firms improve their firm value through the "empowerment" of private placement. At the same time, it has been demonstrated that the pathway through which private placement affect firm value is by increasing operating leverage and reducing financing constraints. However, firm value is also influenced by other factors, and corporate governance plays an irreplaceable role. Wu et al found a significant positive correlation between corporate governance and firm value [29]. Özer et al conducted empirical research on the relationship between corporate governance structure, internal control quality, and firm value [30]. The study found that both internal control quality and corporate governance structure have a positive correlation with firm financial value, and this influence does not significantly differ between state-owned and private enterprises. Corporate governance has a significant promoting effect on value, and value also has a strong feedback effect on governance. To examine the impact of corporate governance on firm value in over-leveraged firms, this paper uses the natural logarithm of the internal control index (*Ic*) as an indicator of corporate governance quality.

*(2) Investment efficiency and firm value*. This paper measures firm investment efficiency by drawing on Richardson’s Expected Investment Deviation Model, with the measurement method as follows.


$${Inveff}_{i,t}=\beta_{0}{+\beta }_{1}{Growth}_{i,t-1}+\beta_{2}{Size}_{i,t-1}+\beta_{3}{Lev}_{i,t-1}+\beta_{4}{Cash}_{i,t-1}+\beta_{5}{Age}_{i,t-1}+\beta_{6}{Roe}_{i,t-1}+\beta_{7}{Inv}_{i,t-1}+\sum Ind+\sum Year+\varepsilon_{i,t}$$

*Inv* represents the ratio of the sum of fixed assets, intangible assets, and other long-term assets to total assets at the end of the year. *Age* represents the number of years a company has been listed. *Roe* represents the profit level, measured using basic earnings per share. *ε* represents the random error term, where a positive *ε* indicates that a company is overinvesting, while a negative *ε* indicates insufficient investment. The absolute value of *ε* is often used to reflect the efficiency of a company’s investment. The closer *ε* is to 0, the higher the investment efficiency, and vice versa. For analysis convenience, this paper follows the approach used by Xie Qiaoxin et al. (2021) in handling investment efficiency and treats the absolute value of *ε* as negative, *Inveff_c* = —| *ε* |. A lower value of *Inveff_c* indicates lower investment efficiency, while a higher value of *Inveff_c* indicates higher investment efficiency.

Table 9 presents the empirical results of the impact of corporate governance and investment efficiency on firm value in over-leveraged firms. The (1) and (2) columns show the regression results for corporate governance and firm value, both of which are significantly positively correlated at the 1% level, indicating that corporate governance has a positive and significant effect on firm value. The (3) and (4) columns show the regression results for investment efficiency and firm value. The results demonstrate that investment efficiency in over-leveraged firms is significantly positively correlated with firm value, suggesting that firms can enhance their firm value by improving investment efficiency.

**Table 9:** Company governance, investment efficiency and corporate value.

variable	(1)	(2)	(3)	(4)
	Evar	Evar	Evar	Evar
Ic	0.180***	0.160***		
	(19.25)	(16.98)		
Inveff_c			0.120***	0.073**
			(3.21)	(1.99)
Cash		0.136***		0.165***
		(9.83)		(9.55)
Size		0.004***		0.008***
		(3.68)		(6.17)
First		0.038***		0.041***
		(4.19)		(3.56)
Soe		-0.015***		-0.019***
		(-5.60)		(-5.78)
_cons	-1.180***	-1.173***	-0.016***	-0.233***
	(-19.48)	(-19.18)	(-8.03)	(-7.75)
Ind	control	control	control	control
Year	control	control	control	control
*N*	3579	3579	2671	2671

*(3) The impact of ownership structure on corporate overleveraging*. To examine the influence of ownership structure on corporate overleveraging, we conducted empirical research with leverage levels (*Lev*) and overleveraging indicator (*Exlevb_dum*) as dependent variables and ownership structure as an independent variable. The results, as shown in Table 10, indicate significant differences in borrowing behavior based on ownership structure. Ownership structure is significantly positively correlated with both *Lev* and *Exlevb_dum*, suggesting that state-owned enterprises tend to have higher leverage levels and are more prone to overleveraging compared to non-state-owned enterprises. Existing studies have found that state-owned enterprises enjoy advantages in borrowing due to factors such as soft budget constraints and government-enterprise relationships [31]. State-owned enterprises, when faced with adverse external conditions and potential adverse consequences of their actions, can rely on external organizations for assistance and continue to survive with less risk exposure. Moreover, due to their government-enterprise relationships, these enterprises may benefit from implicit government guarantees during debt refinancing, resulting in lower barriers to debt refinancing and easier access to financing opportunities. Consequently, state-owned enterprises are more likely to engage in overleveraging.

**Table 10:** Comparison of debt levels between state-owned and private enterprises.

Variable	(1)	(2)	(3)	(4)
	Lev	Lev	Exlevb_dum	Exlevb_dum
Soe	0.130***	0.059***	0.497***	0.356***
	(30.82)	(14.47)	(11.81)	(7.56)
Cash		-0.456***		-4.193***
		(-34.29)		(-22.42)
Size		0.061***		0.032
		(35.58)		(1.63)
First		-0.065***		-0.259*
		(-5.44)		(-1.75)
Ind	control	control	control	control
Year	control	control	control	control
_cons	0.382***	-0.816***	-0.391***	-0.194
	(143.50)	(-21.45)	(-14.21)	(-0.45)
*N*	9437	9437	9437	9437

## 6. Conclusion

The capital structure of a firm has a significant impact on its value. Increasing debt financing can leverage the benefits of financial leverage, lower the cost of capital, and provide tax advantages. However, excessive debt financing can increase financial stress, raise the risk of debt default, hinder debt refinancing, and even limit a company’s growth. This study examined the relationship between a firm’s excessive indebtedness and its value, uncovering the various factors influencing this relationship.

This research find that excessive indebtedness has a negative impact on firm value, with higher levels of overleveraging exerting a stronger inhibitory effect on firm value. Overleveraged firms tend to reduce their debt levels by "increasing equity" through methods such as private placement. Further research revealed that the pathway through which overleveraged firms enhance their value after private placement is by increasing their operational leverage and reducing financing constraints. In terms of influencing factors, the study found that state-owned enterprises have higher levels of debt compared to non-state-owned enterprises. Additionally, among overleveraged firms, improvements in corporate governance and increased investment efficiency can enhance firm value.

In the context of the current structural reform in the supply-side economy, this research sheds light on how overleveraged firms can reduce their debt levels in an orderly and healthy manner, returning to a reasonable capital structure, and ultimately increasing their firm value. The conclusions drawn in this study provide valuable insights for regulatory authorities in their governance efforts. The policy recommendations of this study are as follows: (1) China should adhere to structural deleveraging, especially those enterprises with excessive investment and high debt, and carry out targeted control over the capital flow of these enterprises. (2) The government should guide enterprises to avoid using the way of debt reduction, and take more private placement to leverage, so as to promote the sustainable economic growth of enterprises and enhance the value of enterprises. (3) State owned enterprises should continue to be the focus of deleveraging. As state-owned enterprises play an important role in contributing taxes and stabilizing economic growth, the government should actively explore the path of efficient deleveraging and strictly supervise the asset liability ratio and financial risks of state-owned enterprises. (4) The company should strengthen internal management, focus on the cash flow owned by the enterprise, and avoid the short-sighted behavior of managers for their own interests, which will have a negative impact on the long-term stability and development of the enterprise.

## Supporting information

S1 Data (XLS)
